# A foodborne outbreak of gastroenteritis caused by Norovirus and *Bacillus cereus* at a university in the Shunyi District of Beijing, China 2018: a retrospective cohort study

**DOI:** 10.1186/s12879-019-4570-6

**Published:** 2019-10-29

**Authors:** Dongwan Chen, Yongjin Li, Jinchang Lv, Xiufeng Liu, Peng Gao, Guoxin Zhen, Wenzeng Zhang, Dan Wu, Hongbo Jing, Ying Li, Yao Zhao, Xiaochen Ma, Huilai Ma, Lijie Zhang

**Affiliations:** 1 Shunyi Center for Disease Control and Prevention, No. 1 Guangming South Street, Shunyi District, Beijing, China; 2 Beijing Center for Disease Control and Prevention, Beijing, China; 3 Chinese Center for Disease Control and Prevention, Beijing, China

**Keywords:** Foodborne, Gastroenteritis, Norovirus, *Bacillus cereus*, Outbreak, Cohort study

## Abstract

**Background:**

On September 4, 2018, a boarding school in the Shunyi District of Beijing, China reported an outbreak of acute gastroenteritis. At least 209 suspected students caused of diarrhea and vomiting. The case was investigated, and control measures were taken to prevent further spread.

**Methods:**

A retrospective cohort study was conducted among the school students and staff in order to test hypothesis that high risk of food served at the school canteen. We collected information on demographics, refectory records, person to person transmission by uniform epidemiological questionnaire. Risk ratios (RR) and 95% confidence intervals (CI) were calculated. Stool specimens of cases and canteen employees, retained food, water, and environmental swabs were investigated by laboratory analysis.

**Results:**

We identified 209 cases (including 28 laboratory-confirmed cases) which occurred from August 29 to September 10. All cases were students, and the average age was 20, 52% were male. The outbreak lasted for 13 days, and peaked on September 5. Consumption of Drinks stall and Rice flour stall on September 1 (RR:3.4, 95%CI:1.5–7.8, and RR:7.6, 95%CI:2.8–20.2), Rice flour stall and Fish meal stall on September 2 (RR:4.0, 95%CI:1.2–13.6, and RR:4.6, 95%CI:1.7–12.5), muslim meal stall on September 4 (RR:2.7, 95%CI:1.3–5.4), Barbeque stall on September 5 (RR:3.0, 95%CI:1.2–7.0) were independently associated with increased risk of disease within the following 2 days. Among 35 specimens of rectal swabs or feces from students, 28 specimens were positive. Norovirus GI.6 alone was detected in 23 specimens, *Bacillus cereus* alone in 3 specimens and both norovirus GI.6 and *Bacillus cereus* in 2 specimens. Ten specimens of rectal swabs from canteen employees were positive for norovirus GI, and 2 specimens were positive for *Bacillus cereus*. Four retained food specimens were positive for *Bacillus cereus*, and environmental samples were negative for any viruses or bacteria.

**Conclusion:**

Our investigation indicated that canteen employees were infected by two pathogens (norovirus and *Bacillus cereus*) and transmission may have been possible due to unhygienic practices. Student consumption of food or drink at high-risk stalls was determined as the probable cause of the outbreak.

## Background

Norovirus can be divided into at least 5 genogroups (GI–GV) and at least 35 genotypes. Human disease is primarily caused by GI and GII norovirus. GII viruses are the most frequently detected (89%), whereas GI viruses cause approximately 11% of all outbreaks [1–4]. Since 1999, the most prevalent genotype in mainland China has been GII.4, accounted for 64% of all detected genotypes [5]. In the past decade, most reported norovirus outbreaks were also caused by GII.4, GI norovirus outbreaks were relatively rare, and systematic description of the epidemiology and characteristics of GI outbreaks was even rarer [6]. Yet, this outbreak was caused by the GI norovirus. Norovirus is thought to be the major cause of acute gastroenteritis [7]. Norovirus is highly infectious pathogens that can cause relatively severe disease including vomiting and diarrhea with acute onset. Symptoms usually last up for 1–3 days but can persist longer for young children under 5, elderly, and immunocompromised patients. The average incubation period ranges from 12 to 48 h [8]. Sporadic infections and outbreaks are usually more common in cooler or winter months. Norovirus is transmitted by contact with an infected person or contaminated environmental surfaces, or by eating or drinking contaminated food or water. Per year in the United States, 31 major pathogens caused 9.4 million episodes of foodborne illness, 55,961 hospitalizations, and 1351 deaths. Most (58%) illnesses were caused by norovirus. In Australia norovirus was the leading cause of foodborne illness, accounting for 30% of illnesses caused by known pathogens [9]. While 163 norovirus outbreaks were reported in Japan, foodborne transmission accounted for 58.13% from 2001 to 2005 [10]. In foodborne norovirus outbreaks for which reported the source of contamination, 70% were caused by infected food handlers [11, 12].

On the other hand, *Bacillus cereus* is ubiquitous in nature, such as in plants and soil, in the enteric tract of insects and mammals. Thus, it is easily spread to food products, especially of plant origin, but is also frequently isolated from meat, eggs and dairy products [13]. *Bacillus cereus* causes two different types of food poisoning: the diarrhoeal type and the emetic type. The diarrhoeal type of food poisoning is caused by complex enterotoxins [14, 15], produced during vegetative growth of *Bacillus cereus* in the small intestine [16], and incubation period ranges from 2 to 36 h [8]. While the emetic toxin is produced by growing cells in the food [13], and incubation period ranges from 8 to 16 h [8]. For both types of food poisoning the food involved has usually been heat-treated, and surviving spores are the source of the food poisoning.

On September 4, 2018, a boarding school in the Shunyi District of Beijing, China reported an outbreak of acute gastroenteritis. The boarding school had 5043 students. Totally 20 people became ill on September 4 and 5 with vomiting and diarrhoea. Food from the school canteen was considered as the source of this outbreak. Retained food specimens from the school canteen were tested positive for *Bacillus cereus* on September 6. Meanwhile, Stool samples obtained from 7 students were tested positive for norovirus by real-time RT-PCR. To provide effective control measures, we surveyed the outbreak just verify additional cases, source of infection, vehicle for infection, and mode of transmission.

## Methods

### Study design

When the outbreak was reported, an investigation began immediately. Because of the investigation was in response to a public health emergency, and section 108 of Food Safety Law of the People’s Republic of China law (chapter 7) provides that, after a food safety incident occurs, the investigation department have the right to find out from relevant units and individuals about the situation related to the incident, and collect relevant information and samples. The relevant units and individuals shall cooperate with them and shall not refuse. Thus the disposal of outbreak was exempted from ethical approval and does not require informed consent. In spite of this, we still gave oral announcement to all respondents before the investigation. Ultimately we obtained a part of signed questionnaires, part of subjects by telephone interview. A questionnaire survey (see Additional file 1) includes basic personal information (name, gender, age, class, dormitory etc.), date of illness onset, duration of illness, clinical symptoms, treatment, and history of exposure to suspected food, water and patients. Moreover, we also collected the refectory records from all students and compared between cases and non-cases. All investigation data will be filed by Shunyi Center for Disease Control and Prevention, and not be disclosed to third parties.

A retrospective cohort study was conducted among school students and staff to test the risk hypothesis in canteen. Canteen manager provided refectory records of 33 food stalls from August 31 to September 8. During the period from September 1 to 5, exposure to high risk foods or patients was most likely to explain the symptoms that occured from September 1 to 7 (assuming an incubation period of 2–48 h). Exposures prior to August 31 were not included in the exposure investigation as this was the summer vacation during which only few students in school. Based on daily food exposure to asymptomatic person, we established 5 crowd cohorts from September 1 to 5. Over the next 2 days, the number of postprandial cases at different stalls was divided by the total number of diners at the stall, to calculate the attack rate (AR). For each specific date (September 1, 2, 3, 4 and 5), by comparing the dining status between case and non-case in different stalls, to calculate risk ratios (RR) and 95% confidence intervals (CI). The data were inputted by EXCEL v2010 (Microsoft) and analyzed by SPSS v25.0 software (SPSS Inc., Chicago, IL, USA). *p*-value was two-sided and *p* < 0.05 was considered statistically significant.

### Case definition

The investigated subjects included students and staff in the university. Suspected case was defined by the onset of vomiting or diarrhoea (≥3 times per day) in the university since August 27, 2018. Laboratory confirmed case was the stool or vomit specimen of suspected case tested positive for *Bacillus cereus* or norovirus.

### Case finding

We collected case information from nearby hospitals, school infirmary, and head teachers, with a special focus on patients with vomiting, abdominal pain, diarrhoea, and fever. Each case was confirmed either by face-to-face questionnaire or by telephone.

### Laboratory and environmental investigations

We collected rectal swabs, feces and vomit samples from cases, rectal swabs from canteen employees, retained food samples, and drinking water samples including tap water and self-providing well water. Detection of norovirus by reverse transcription polymerase chain reaction (RT-PCR). Viral RNA was extracted from 140 μL of fecal sample diluted 1:10 in 0.05 mol/L phosphate-buffered saline using the QIAamp Viral RNA Mini Kit (QIAGEN, Hilden, Germany), according to the manufacturer’s instructions. RNA was stored at − 20 °C until further use. The One-Step RT-PCR Kit (QIAGEN, Hilden, Germany) was used to amplify norovirus genes in the open reading frame 1 (ORF1) and in the junction gene region between ORF1 and ORF2. Targeted to the regions B and C of the norovirus genome, a novel RT-PCR assay was performed with primers MON432/G1SKR for GI viruses and primers MON431/G2SKR for GII viruses, yielding 579 bp and 570 bp PCR products. The RT-PCR products were sent to Thermo Fisher Biochemicals (Beijing) Ltd. (Beijing, China) for sequencing using an ABI 3730xL DNA Analyzer (Applied Biosystems, Foster City, CA, USA). Intestinal bacteria including *Bacillus cereus*, Salmonella, *Staphylococcus aureus*, Proteus spp., *Vibrio parahaemolyticus* and pathogenic *Escherichia coli* was confirmed by culture. Total bacterial count, total coliforms, thermotolerant coliforms and norovirus were detected in water samples. The culture medium was mainly provided by Beijing Land Bridge Technology Co., Ltd. (Beijing, China).

## Results

### Descriptive epidemiology

**Fig. 1:**
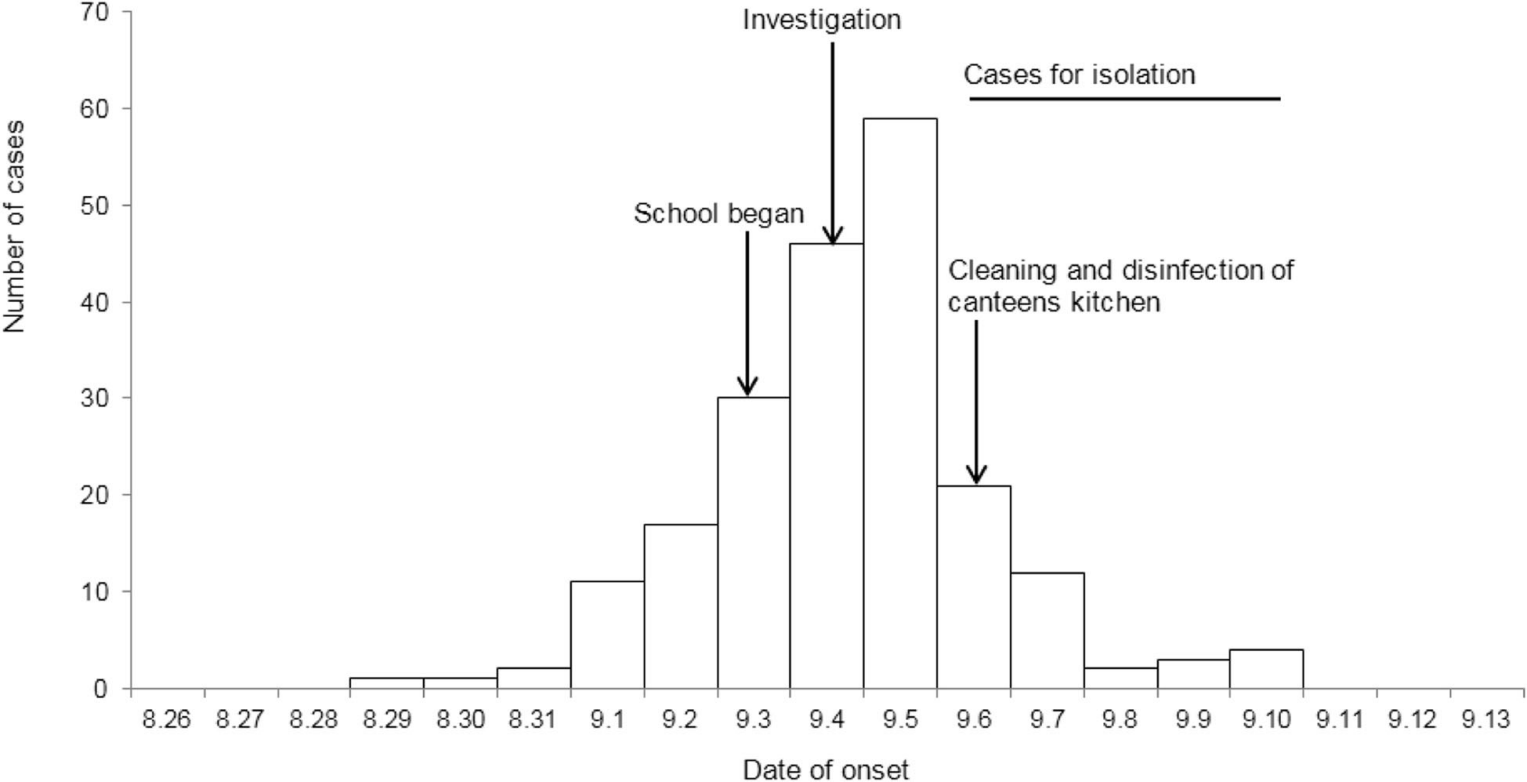
Epidemic curve for probable outbreak cases of acute gastroenteritis by 24 h intervals at a university—Shunyi, Beijing, China, 2018

We identified 209 suspected cases between August 29 and September 10, 28 of which were laboratory confirmed cases. All cases were students and age ranges from 17 to 23. The incidence rate was 4.1% (209 / 5043). The school is a boarding school, and cases were widely distributed in all 6 dormitory buildings and also in 118 classes. No aggregation of dormitories and classes was found. In addition, no cases were found among faculty members and their families, and no similar cases were found among urban residents around the school. The main clinical symptoms of the cases were nausea (70.3%), diarrhea (69.9%), vomiting (65.6%), abdominal pain (64.1%), fatigue (40.2%), dizziness (36.4%), fever (29.7%), and headache (24.9%). There were no cases of hospitalization or death. Fifty-two percent (108/209) of the cases were male, and the median age of onset was 20 years (range 17–33 years). The outbreak lasted 13 days from 17:00 on August 29. The high-peak occurred on September 5 and no further cases were reported after September 10 (Fig. 1).

### Analytical epidemiology

In order to verify that dining in the canteen was a high risk factor, we analyzed people who ate in canteen on any day from September 1 to 5, to determine the relationship between consumption of different stalls and onset. The selected cases were those who developed the disease within 2–48 h after dining in the canteen. The incidence of daily diners from September 1 to 5 was 2.1, 1.5, 2.3, 2.4 and 1.5% over the next 2 days. Cohort study found that multiple high-risk stalls were present every day on September 1, September 2, September 4, and September 5, suggesting that those who had eaten in those stalls were at greater risk of developing the disease in the next 2 days. We didn’t find any high-risk stalls on September 3 (Table 1).

**Table 1:** Daily attack rates and risk ratios of gastroenteritis among students having meals on high-risk stalls of the school canteen, September 1–5

Date	High-risk stalls	Exposed			Non-exposed					
		Total	Cases	AR%	Total	Cases	AR%	RR	95% CI	P
September 1	Drinks	299	13	4.3	777	10	1.3	3.4	1.5–7.8	0.002
	Rice flour	48	6	12.5	1028	17	1.7	7.6	2.8–20.2	0.000
September 2	Rice flour	53	3	5.7	1826	26	1.4	4.0	1.2–13.6	0.046
	Fish meal	81	5	6.2	1798	24	1.3	4.6	1.7–12.5	0.003
September 4	Muslim meal	152	9	5.9	3468	77	2.2	2.7	1.3–5.4	0.008
September 5	Barbeque	152	6	3.9	3059	41	1.3	3.0	1.2–7.0	0.023

*AR* attack rate, *RR* risk ratio, *CI* confidence interval

### Laboratory inspection

Among 35 specimens of rectal swabs or feces from students, 28 specimens were positive (positive rate 80%). Norovirus GI.6 alone was detected in 23 specimens, *Bacillus cereus* alone in 3 specimens and both norovirus GI.6 and *Bacillus cereus* in 2 specimens. Rectal swabs from 124 canteen employees were tested on September 6, and 10 were positive for norovirus GI.6 (positive rate 8.1%). In addition, rectal swabs from 2 canteen employees were positive for *Bacillus cereus*, and one of them worked in the Barbeque stall, which a high-risk one. Of the seven retained foods, four were positive for *Bacillus cereus*, and the test results were in the range of 10 to 1.6 × 10^5^ CFU/g. Norovirus was not detected in retained foods. Seven environmental samples and six drinking water samples for bacteria and norovirus tested negative.

### Environmental hygiene investigation

Students ate only in the student canteen, which had 33 stalls. Each of the food stalls operated independently, with different types of food. On September 4, investigation found that the food raw materials and public tablewares in the kitchen operation of some stalls were disorderly, and the sanitary conditions were poor. Some of the food was stored at room temperature for a long time. The water supply mode of the school was self-built facilities water supply, the water supply sanitation license was effective, the disinfection equipment was running normally, and the water supply process met the relevant specification requirements. There were two self-supply wells in the school, no pollution sources within 30 m around the wells, and the sanitary protection of the wells accorded with the requirements. Drinking water mode was mainly drinking brand of bottled water and boiled water heated by electric water heater. According to the investigation of the canteen employees showed that 3 norovirus-positive employees reported symptoms of nausea, diarrhoea and vomiting between September 1 and 2, and continued to work until September 6. All norovirus-positive employees were closely connected, even after someone developed symptoms. They shared the toilet next to canteen, and some lived together and ate high-risk foods, such as rice flour or fish meal, made by each other (based on a survey of four people’s eating history). Eventually, their work stalls had become high-risk (such as Drinks, Rice flour and Fish meal stalls).

## Discussion

The outbreak of acute gastroenteritis, which affected 4.1% of the population of a boarding school, was likely caused by norovirus and *Bacillus cereus*. The reason for the low incidence of norovirus might be that it was limited to foodborne transmission in the short term and had not developed to the late stage of person to person transmission. Due to the appropriate measures in the later stage, norovirus had not spread completely on campus. In addition, there were only a few high-risk stalls in the canteen every day, it might also be the cause of no more cases.

93.8% of the cases occurred in 7 days from September 1 to 7, and the outbreak lasted almost 2 weeks. The epidemic curve showed that the onset time was concentrated, which strongly indicated that the outbreak was continuous exposure pattern rather than person to person transmission pattern. In addition, the lack of spatial aggregation of cases also indirectly supported food-borne transmission. At the same time, due to students’ resistance, early isolation of student cases was not ideal. But after the implementation of measures such as closure of high-risk stalls, isolation of canteen employees with norovirus and *Bacillus cereus* positive, and thorough disinfection of canteen, the outbreak quickly subsided. This further confirmed the hypothesis that food supplied in the canteen was the root cause of the outbreak. Through retrospective cohort study, daily cohort was established and personnel exposure in each stall every day was calculated. Finally, multiple high-risk stalls were found, which provided strong epidemiological evidence for food-borne transmission. Because the outbreak was continuous exposure, this approach reduced the chance that cases might be misclassified and ensured that they were placed in a cohort every day as non-sick people.

Laboratory evidence suggested that the positive rate of norovirus was 71.4% (25/35) among the cases. Although this rate may be over-estimated due to testing on only some selected cases, it was still much higher than the prevalence of norovirus reported in patients (17%) with acute gastroenteritis in developing countries [17], supporting the causative role of norovirus in this outbreak. Moreover, our study is the first reported outbreak of genotype GI.6 norovirus in the Shunyi District of Beijing, China. Compared with non-GI.6 outbreak, the GI.6 outbreak was characterized by food-borne transmission [18]. When canteen employees served in these high-risk stalls, food might be contaminated by their faeces, or unhygienic practices by employees who expelled viruses and bacteria. Viruses and bacteria could also be excreted without symptoms, so improper handling by asymptomatic food handlers could also lead to outbreaks. *Bacillus cereus* had also been detected in the outbreak, which is believed to be an opportunistic pathogen that causes gastrointestinal symptoms associated with the production of cereulide (emetic toxin) or enterotoxin (diarrheal syndrome). In this outbreak, between 10 and 1.6 × 10^5^ CFU/g *Bacillus cereus* were detected in the incriminated foods, and diarrheal or emetic syndrome was often associated with *Bacillus cereus* counts of 10^5^ to 10^8^ cells or spores [19]. In addition, *Bacillus cereus* was detected in stool samples of canteen employees and cases, and one of employees who tested positive sold *Bacillus cereus*-positive foods.

Based on the experience of the outbreak, the following suggestions are put forward. Firstly, preventive measures such as cases for Isolation, health education, cleaning and disinfection of dwelling and dining place, identification and exclusion of symptomatic food handlers, even if the cause of the disease is not clear, should be carried out at an early stage, to avoid further development of the outbreak. Secondly, epidemiological investigations need to be carried out in a timely manner, especially in this outbreak caused by norovirus, transmission of the virus is diverse, and it is very difficult to detect the virus in contaminated food because of the low titer of virus. So, to identify risk factors, it is all the more important to use epidemiological investigation. Finally, in order to quickly identify pathogens that cause for outbreaks, samples still need to be submitted at the same time for pathogen detection, and to implement targeted control measures.

There were also some limitations in this study. Firstly, it was a large-scale outbreak, but the number of investigators was limited. This had led to some cases not being verified and underestimating the incidence of the population. Secondly, only the existence of high-risk stalls was investigated, and the information on food manufacture and sale could not be obtained. As a result, this made it impossible to analyze key control links in the processing and storage of food. Finally, some canteen employees might have failed to reveal any symptoms of gastroenteritis to the investigation team due to fear of adverse consequences, but this information was important to further trace the source.

## Conclusion

Our survey showed that canteen employees were infected by two pathogens (norovirus and *Bacillus cereus*) and transmission may have been possible due to unhygienic practices. Student consumption of food or drink at high-risk stalls was determined as the probable cause of the outbreak. Epidemiological investigation proved to be useful in determining the probable source of the infection and to implement timely intervention measures.

## Supplementary information

**Additional file 1.** The self-compiled case questionnaire used in this survey. The main purpose of this questionnaire is to obtain the basic situation of case and the related risk factors of the outbreak, such as meal situation, contact history, etc. The contents of the questionnaire can be obtained in the Figshare repository https://figshare.com/articles/Additional_file_1/8015762.

## Data Availability

The data that supports the conclusion of this article is available in the Figshare repository https://figshare.com/articles/dataset_xlsx/7945094.
