# Supplementary material for: A foodborne outbreak of gastroenteritis caused by Norovirus and Bacillus cereus at a university in the Shunyi District of Beijing, China 2018: a retrospective cohort study
Source: BMC Infect Dis. 2019 Oct 29;19:910. doi: 10.1186/s12879-019-4570-6 (PMC6819576; doi:10.1186/s12879-019-4570-6)
Supplement: Supplementary file 1 — Additional file 1. The self-compiled case questionnaire used in this survey. The main purpose of this questionnaire is to obtain the basic situation of case and the related risk factors of the outbreak, such as meal situation, contact history, etc. The contents of the questionnaire can be obtained in the Figshare repository https://figshare.com/articles/Additional_file_1/8015762. [file 12879_2019_4570_MOESM1_ESM.doc]

Food-borne Diseases Case Questionnaire

**1. Basic personal information**

Name_______ Gender __ age __ Residential address_____________________

Work unit___________________ Unit address____ ______

Contact phone ___ Location of investigation___ __

Investigation time Year Month Day Hour Minute

**2. Sings and symptoms**

2.1 First symptom____ _ Onset time month Day Hour Minute (24 hours system, the time when first symptoms appear)

2.2 Clinical manifestations: order of occurrence of symptoms__________________

2.2.1 General symptoms and signs (tick in the appropriate box)

Temperature ____ ℃ □chills □dizziness □headache □cough □other (details)________

2.2.2 Digestive tract symptoms and signs (tick in the appropriate box)

□nausea □vomit times/24 hour □bellyache □upper abdomen □lower abdomen □umbilical week

□colic □labor pain □dull pain

□diarrhea____ times/24 hour □lean stool □pasty □watery stool □mucus stool

□purulent blood stool □meat washing water stool □rice-water stool

□tenesmus □other (details)__

2.2.3 Neurological symptoms and signs (tick in the appropriate box)

□convulsions □dizziness □blurred vision □coma □delirium □speech difficulties □dysphagia □dyspnea □diplopia □epilepsy □pupil changes (□enlargement □fixation □contraction) □other (details)____

2.2.4 Special symptoms and signs (tick in the appropriate box)

□Jaundice □lymph node swelling □neck joint stiffness □thirst □cyanosis _ site □flush across the cheeks □metallic taste □pigmentation □other (details)

2.2.5 Other symptoms___

**3.** R**elevant information** (tick in the appropriate box)

3.1 People crowding around (including recent relatives and friends) have similar symptoms

□No □How many people? □Unknow

3.2 Family members have similar symptoms □No □How many people? □Unknow

3.3 History of allergy to food □No □Yes, Allergic food is □Unknow

3.4 Recent travel history

3.5 Pet keeping__ 3.6 Situation for drinking water_______ 3.7 other ____

**4. Treatment situation**

□No treatment □Hospital treatment, medical unit___________________ clinical diagnosis______

Medication situation (drug name, dose and medication time)_________ □Self-medication (drug name and dose)______

**5.** **Clinical and laboratory test results** (no clinical or laboratory test result, you may not fill in this item)

Specimen type Collection time Number Laboratory test results

5.1 Complete blood count

5.2 Stool routine

**6.** **Eating survey (food intake questionnaire within 72 hours before onset)**

| Eating condition | The day of onset( month day) | | | | | | the day before the onset( month day) | | | | | | two days before the onset( month day) | | | | | |
| --- | --- | --- | --- | --- | --- | --- | --- | --- | --- | --- | --- | --- | --- | --- | --- | --- | --- | --- |
|  | breakfast | | lunch | | dinner | | breakfast | | lunch | | dinner | | breakfast | | lunch | | dinner | |
|  | species | quantity | species | quantity | species | quantity | species | quantity | species | quantity | species | quantity | species | quantity | species | quantity | species | quantity |
| Name and quantity of food |  |  |  |  |  |  |  |  |  |  |  |  |  |  |  |  |  |  |
|  |  |  |  |  |  |  |  |  |  |  |  |  |  |  |  |  |  |  |
|  |  |  |  |  |  |  |  |  |  |  |  |  |  |  |  |  |  |  |
|  |  |  |  |  |  |  |  |  |  |  |  |  |  |  |  |  |  |  |
|  |  |  |  |  |  |  |  |  |  |  |  |  |  |  |  |  |  |  |
|  |  |  |  |  |  |  |  |  |  |  |  |  |  |  |  |  |  |  |
|  |  |  |  |  |  |  |  |  |  |  |  |  |  |  |  |  |  |  |
|  |  |  |  |  |  |  |  |  |  |  |  |  |  |  |  |  |  |  |
|  |  |  |  |  |  |  |  |  |  |  |  |  |  |  |  |  |  |  |
|  |  |  |  |  |  |  |  |  |  |  |  |  |  |  |  |  |  |  |
| time |  | |  | |  | |  | |  | |  | |  | |  | |  | |
| source |  | |  | |  | |  | |  | |  | |  | |  | |  | |
| dining place |  | |  | |  | |  | |  | |  | |  | |  | |  | |

Other suspicious foods ___ food source ___ eating time ___ eating place ___ quantity ___

Signature of the person under investigation (or his guardian) ___ Identity of guardian _  ____Year Month Day

Investigator signature ___ _ _ _ _ _ ____Year Month Day
